# Environment and Operation

**Published:** 1910-11-19

**Authors:** 


### SURGERY.

### ENVIRONMENT AND OPERATION.

The progress of bacteriology and the evolution of aseptic technique have so altered the whole of surgical art that, in considering the indications for many operations, not merely the condition of the patient but also the condition and surroundings of the surgeon have to be taken into account.

The operations now done on the human body have been greatly increased by the addition of a large number in which manual dexterity is relatively of slight value compared with aseptic technique, and in which this latter has become a sub-conscious part of the surgeon who practises it as a habit without continually thinking of it.

### Types of Operation.

For this reason a large number of operations are barred to the general practitioner, who may be perfectly competent to do them so far as skill with the fingers is concerned, but who has so few cases that the armamentarium of an aseptic technique is a profitless investment and its practice not sufficiently often used to develop into a habit. Considered from this point of view all operations may be put into three classes:—

1. Cases where immediate operation is necessary to save the patient's life.
2. Cases where immediate operation is not necessary, but where one may be advisable to save the life of the patient in the future.
3. Cases where operation is not essential so far as life is concerned, but where it is advisable, either to ward off possible danger in the future or to raise the wage-earning capacity of the individual; that is, where an operation is a form of insurance to the patient.

### Operations of Urgency.

In the first class may be included all cases of peritonitis, whether from appendicitis, from perforated ulcers, or any other cause; the relief of retention of the urine; the surgery of many injuries, such as ruptured viscera, meningeal hæmorrhage, and penetrating wounds; cæsarean section, operations for strangulated hernia, and tracheotomy.

Under the second come all operations for malignant disease, operations for acute septic processes, and for gangrene or for tuberculous disease.

In the third class may be put all radical operations for hernia, for hydrocele, for varicocele, all osteotomies, the surgery of paralyses and deformities, and the operative treatment of simple fractures.

In this last class of case surgical technique is of the very highest importance; if it fail, not only is no good done, but usually irreparable harm is caused. To convert a simple fracture into a septic compound one involves a terrible responsibility. To allow a radical operation for hernia to suppurate robs the individual of all chance of raising his wage-earning capacity, for not only is the hernia likely to recur, but it will probably suppurate again if operated on a second time.

### The Question of Asepsis.

For these reasons the general practitioner is probably very wise not to operate on this class of case, unless he can feel that his aseptic technique is as much above criticism as is that of the hospital surgeon.

We do not mean to imply by this that asepsis is not equally important in the second class of operations, but the responsibility of the surgeon is not so great as in the third class of case. The malignant disease would have killed anyhow, the tuberculous abscess would have broken down and become infected. The surgeon has here merely anticipated Nature, not added a new risk which was not there before.

But when we turn to the first class of cases the matter is different. Asepsis is still of paramount importance towards success in treatment, but the dangers of leaving the patient are so great that the absence of the armamentarium of aseptic technique must not be weighed against the urgency of the operation to save the patient's life. The surgeon must do the best with such as he has at hand. Country hospitals are so numerous, and motor-cars so swift, that the number of cases where a general practitioner is face to face with operation or loss of his patient are fewer and fewer. But they do still occur, and everyone must be prepared to "tackle" such an emergency.

### Asepsis versus Antisepsis.

When such is the case an antiseptic rather than aseptic process will probably be the best. The great difficulty is to obtain cold sterile water. The only means of sterilising is by boiling, and the time this takes to cool down to allow of the fingers being dipped in it to remove instruments or to scrub up the patient is very considerable. When such an emergency case is to the fore, all the available kettles should be started boiling and the water when sterile poured off into other receptacles to cool. Then if there is an hour or two between the decision and the operation, sufficient cold sterile water will be forthcoming to mix with fresh-boiled water to make up lotions or to use for sterile saline solution.

The interior of basins, etc., may be rapidly and efficiently sterilised by putting in a few drops of methylated spirit and setting light to it. The inside of jugs and cans are best rinsed out with boiling water, which is thrown away before the jug be filled.

### Iodine Sterilisation.

For the sterilisation of the skin a long period of compresses before the operation is no longer considered necessary; to paint the skin area with a 2 per cent. solution of iodine in rectified spirit a quarter of an hour before the operation, and then again when the patient is under the anæsthetic is all that is necessary. Care must, however, be taken that the skin has not been washed with soap and water just before its application; and therefore any shaving of pubic hairs, etc., must be done some quarter of an hour before the first painting. The surgeon's hands may be treated in the same way, the margins of nails and folds of skin being painted with iodine solution, and this allowed to soak in before the scrubbing with soap and water.

### Conclusions.

We see then three things. Firstly, that in discussing the pros and cons of every operation the surgeon and his surroundings must be considered as well as the local and general condition of the patient. Secondly, that there is a large, a very large, number of operations which no surgeon should consider himself justified in performing unless he can trust his own aseptic technique, and unless he has the patient in surroundings where that technique can be carried out. Thirdly, there is a class of operations where the surgeon must consider the question of doing the operation himself, hampered by inefficient surroundings for an ideal technique, against the risk of moving the patient for a distance to some hospital, and where he must be prepared to operate himself if he decide that this gives the patient the better chance. Further, when he does operate he must do his best to imitate the surroundings of a hospital operating-theatre by methods of which we have noted one or two points above.

